# Notes and News

**Published:** 1890-05-10

**Authors:** 


### Notes and News.

Collected and Communicated.

On April 18th a meeting was held at Shipston-on-Stour to consider the proposal to establish a cottage hospital. Unfortunately no very favourable site seems to be available. After some discussion it was resolved, on the proposition of the chairman, seconded by the Rev. C. P. Causton, "That this meeting is of opinion that it is desirable to establish a cottage hospital in Shipston or its immediate neighbourhood, provided the necessary funds can be obtained." The following persons were appointed a committee, with power to add to their number, viz.: Miss Dickins, Mrs. Sheldon, Messrs. C. Stein, M.D., J. Eden Hiron, J. C. Greenhill; and the committee pledged itself to get donations and annual subscriptions, and to make further inquiries on the subject generally as they may deem necessary.

Some disappointment was caused at the annual meeting of the London Homœopathic Hospital by the absence of Lord Ebury through illness; however, Major Vaughan Morgan efficiently filled the chair. The annual report showed that the increase of in-patients had been 118 more than in any previous year, the total amounting to 830, while the out-patients had numbered 10,363, against 9,486 in the previous year. The current income had been £4,706, and the expenditure £4,579, leaving a small balance which had been partly devoted to the repayment of the debt of previous years to the reserve fund. Miss Barton had founded "The Mary Bed," and endowed the "Barton Bed" at a cost in each case of £1,000, while Miss Isabella Barton had founded the "Alice Cot" by a gift of £750. The post of patron of the hospital had been accepted by the Princess Mary (Duchess of Teck). With regard to the proposed rebuilding of the hospital, nearly £6,000 had been promised, and the board hoped soon to see their way to embarking on what must be a long and arduous effort. Major Vaughan called the attention of those present to the fact that a concert would be held at Grosvenor House on June 5th in aid of the institution, and claimed that all the great singers were homœopaths. This is rather too sweeping an assertion.

The Lord Mayor is doing his work nobly, so far as taking the chair at charitable dinners is concerned, and we are glad to see he will preside on May 12th at the festival dinner of the Association for the Oral Instruction of the Deaf and Dumb. This Association owes its origin to the benevolence and perseverance of the late Baroness Mayer de Rothschild. The success which attended the first introduction into England of the German system (teaching by means of Speech and Lip-reading), by Mr. Van Praagh, at the Jews' Deaf and Dumb Home, was so great, that she determined to extend its blessings to the afflicted of every race and creed, and to establish a Training College for Teachers. One of the most curious and instructive sights in London can be seen at the college every Wednesday afternoon, when a public lesson in Lip-reading is given to pupils in various stages of instruction.

We mention the pictures at the New Gallery last week; at the Academy are portraits of Dr. William Travers, Dr. J. Birkbeck Nevins, Sir Oscar Clayton, by Frederick Goodall, R.A.; and Charles B. Keetley, F.R.C.S., by Seymour Lucas. An excellent portrait of Sir Edmund Hay-Currie, by John Pettie, R.A., will also interest our readers, and an oil painting of F. Cleeve, Esq., C.B., late Chairman of the Seamen's Hospital Society. Amongst the miniatures is a tiny head of Dr. Douglas Pidcock, and in the Black-and-White Room is a delightful drawing of Dr. Lander Brunton, done by Lowes Dickinson. There is also a marble bust of Sir William Savory and a wax medallion of Dr. Charcot in the Lecture Room. At the Grosvenor Gallery there is a marble bust of Dr. G. Johnson, Physician Extraordinary to the Queen; a bust in bronze of Dr. Frank Corner, and a very exquisitely wrought marble relief of Mr. Henry C. Burdett by Waldo Story.

Among those present at the meeting of the Hospitals Association on April 30, in the Governors' Hall of St. Thomas's Hospital, were:—The Right Hon. the Earl of Meath (in the chair), Mr. Thomas Ryan (Secretary of St. Mary's Hospital), the reader of the paper on "London's New Street Ambulance Service," Dr. Bristowe (the President), Mr. W. J. Church Brasier (Deputy Supt., Invalid Transport Corps, St. John Ambulance Association), Dr. Mitchell Bird, Mr. Henry C. Burdett, Superintendent Cutbush (Scotland Yard), Mr. Alfred Carter, Miss Culverhouse, Mr. John Furley (Hon. Director of Stores, St. John Ambulance Association), Mr. H. Howgrave Graham (Secretary, Hospital for Epilepsy), Miss Gordon (Matron, Charing Cross Hospital), Miss Griffiths (Matron, Lambeth Infirmary), Mr. Percy Jago, Dr. Lees, Miss Medhill (Matron, St. Mary's Hospital), Mr. P. Michelli (Secretary Seamen's Hospital, Greenwich), Mr. Donald Mackenzie, Rev. Watson King Ormsby, Mrs. Phillips (Matron, Queen Charlotte's Hospital), Major Sir Herbert Perrott, Bart. (Chief Secretary, St. John Ambulance Association), Surgeon-Major W. H. Platt, Mr. G. Owen Ryan (Secretary Queen Charlotte's Hospital), Sir Edward H. Sieveking, Dr. Steele (Superintendent, Guy's Hospital), Mr. J. S. Wood (Secretary of Olympia), and Mr. Mark Whitwell (Treasurer, Bristol Hospital for Sick Children).

Mr. Ryan stated that only a year ago the scheme for a complete ambulance service for the metropolis was first started. There were then but a few ambulances scattered irregularly over the metropolis. Now, though their work of supplying the stations was but half completed, there were 39 ambulance stations already equipped, 20 of which were at stations of the Metropolitan Fire Brigade, 12 at hospitals, 3 at buildings of the Industrial Dwellings Company, 3 on cab ranks, and 1 at Holborn Viaduct. With regard to the ambulances themselves, a great deal of trouble has been taken in deciding upon the best form of litter for the work, with the result that one made by Mr. Carter, of Holborn Viaduct, improved and strengthened for the purpose, has been adopted. Attached to each ambulance was a basket containing splints, bandages, and other requisites for rendering "first aid" to the sufferer.

The following vacancies are announced this week, the amount of the salary in each case being given after the name of the institution :—

**Resident Medical Officers.**

Stirling District Asylum, Larbert, Assistant Medical Officer—£100, board, etc.

Lancaster Infirmary, House Surgeon—£80, board, etc.

Barnwood House Hospital, Gloucester, Junior Assistant Medical Officer—£100, board, etc.

Royal Hospital for Diseases of the Chest, House Physician—£40, board, etc.

North London Consumption Hospital, Medical Officer—£40, board, etc.

Kent County Asylum, Second Assistant Medical Officer—£120, board, etc.

Hants County Asylum, Third Assistant Medical Officer—£100, board, etc.

Wirral Children's Hospital, House Surgeon—£50, board, etc.

Holloway Sanitorium, Clinical Assistant.

**Non-Resident Medical Officers.**

Plymouth Hospital, three Honorary Assistant Surgeons.

Dunmow Rural Authority, Medical Officer of Health—£50.

Viscount Cross presided at the annual meeting of the Grosvenor Hospital for Women and Children. From the report it appeared that during the past twelve months 103 women were admitted as in-patients, in addition to twelve who were in the wards on the 1st January, 1889, making a total of 115 in-patients for the year, against 89 in the year 1888. The number of out-patients during the year amounted to 2,232, against 2,285 in 1888, the total number of attendances being 9,716, against 9,332. The income for the year amounted to £1,082 7s. 8d., against £1,135 17s. 2d. ; and the expenditure to £1,115 13s. 3d. against £1,140 9s. 9d. The extension fund, including sums promised, now amounts to £1,727 14s. 9d. The "fancy fayre" held at the Royal Albert Hall in May last yielded £209 5s. 2d.

The Metropolitan Hospital, situated in the East-end of London, the only one in a district containing a quarter of a million inhabitants, and a hospital at which the Provident System has been successfully at work for considerably over two years, held its festival dinner at the Whitehall Rooms, Hôtel Métropole, on Tuesday. The Right Hon. the Earl of Derby, K.G., occupied the chair, and was supported by Sir W. Guyer Hunter, M.P., Mr. Cyril Flower, M.P., Mr. Joseph Fry, and about a hundred others. In the course of his speech in proposing "Prosperity to the Hospital," Lord Derby, referring to hospital abuse, said that an endeavour was being made to remedy this in the Metropolitan Hospital by means of the Provident System, by which those who could pay, even were it only a little, were asked to contribute towards the cost of their medical relief. This principle was exposed to a severe test, for that hospital asked people to pay in part for that which they had been accustomed to get for nothing in the past, and which they could get for nothing in other parts of London. The experiment, however, as far as it had gone, had been entirely satisfactory. Last year it was stated that 11,000 persons had placed themselves under the new arrangement, while this year the number had increased to over 16,000.

Mr. Cyril Flower, M.P., in the course of his reply to this toast gave some interesting figures as to the proportion of beds in hospitals to inhabitants in our larger towns. In London there was on an average one bed to about every 700 inhabitants; Birmingham, one to 1,000; Bradford, one to 900; Liverpool, one to 740 ; Bristol, one to 500; Norwich, one to 400; Leeds, one to 320; and Canterbury, one to 200. In the Metropolitan Hospital, however, the proportion was, one bed to every 3,300 persons in that district; a state of things which, the speaker said, should not be allowed to exist much longer. Subscriptions amounting to nearly £1,800 were announced by the secretary (Mr. Charles H. Byers).

At Marlborough the question of an infectious diseases hospital is to the fore. There has lately been a slight outbreak of diphtheria in the town, and the cottage done up by the Rural Sanitary Authority can only take two cases. There is, however, an iron hospital at Marlborough which might suffice, if the Town Council will act in concert with the Rural Sanitary Authority, and so save the ratepayers' pockets.

The particular claim which Mr. Blundell Maple is to advocate at the dinner on May 21st, in aid of the North London Consumption Hospital, is in furtherance of an extension to the present building, by the erection of a central block, which will provide a new entrance-hall, a patients' dining-hall, with other offices, and accommodation for thirty additional indoor patients. Already £2,000 has been promised by members of the committee, but all desire to largely increase this sum.

In London the last elections have given us an increase of eight in the number of lady guardians. In the country also the ladies have been very successful, and the result will be welcome to all thoughtful people. But a great responsibility rests on a woman who takes a public position, and they are often devoid of that training and that knowledge of public affairs in which every man is reared. Therefore, it is to be hoped that all lady guardians will walk cautiously and not try too sweeping reforms, nor yet be laggards when work is to be done. The care of the sick and the state of the union infirmaries we specially commend to the notice of all lady guardians.

The forty-first annual report of the Royal Portsmouth, Portsea, and Gosport Hospital for the year ended December 31st, 1890, says that, since the opening of the improved hospital in May, 1888, the wards have been furnished with 20 additional beds, and the institution now contains (including the 30 beds in the Lock department) 125 beds for the reception of patients. This important increase has, however, been gradually introduced, and, fortunately, by the help of certain improvements in the administration, without any serious financial strain. The total number of patients treated in 1889 exceeded by 184 that of the previous year. The Committee, after acknowledging the valuable services of the matron (Miss Tillett), state that the payment for the nursing sisters has largely extended, and no less than 95 cases have been attended by the eight competent nurses engaged in this work. The funds of the institution have derived a profit, including training fees of £302 during the year from their services, a sum equal to the amount paid for the salaries of the whole of the nursing staff of the hospital.

We are all beginning to make plans for the summer now, and to look forward to that short rest in some health locality which even the busiest of us must take. And the friends of the poor children are not forgetting their charges. There was a very large gathering at the Charterhouse last week on the occasion of the annual meeting of the Children's Fresh Air Mission, presided over by Canon Elwyn. The object of the mission is to give poor children, taken mainly from the districts of Holborn, Clerkenwell, and St. Luke's, a fortnight or three weeks' holiday in fresh air, preference being shown to those who are in feeble health, or who could not otherwise obtain an outing. Last summer 2,623 children thus enjoyed the benefits of the mission, and during the last eight years no fewer than 12,206 children have been benefited. The Children's Country Holidays Fund, 10, Buckingham-street, Strand, sent away over 20,000 children last year for the fortnight of fresh air and cottage life which they need so much. There is no sign that the country "mothers" are getting tired of the children, and the London mothers, we understand, have contributed a very large proportion of the cost themselves—a proportion which increases by a fraction every year. Other agencies are also at work, and it is to be hoped for the sake of the general health of our great towns that these agencies will be well supplied with funds.

